# Multimodal Neuroimaging and Electrophysiological Markers in Multiple Sclerosis: An Integrative Review of fMRI, EEG, and EMG Approaches: fMRI, EEG, EMG in Multiple Sclerosis

**DOI:** 10.31661/gmj.v14i.3878

**Published:** 2025-10-11

**Authors:** Mohammad Hossein Salemi

**Affiliations:** ^1^ Department of Psychology, University of Tehran, Iran

**Keywords:** Multiple Sclerosis, Functional MRI, Electroencephalography, Electromyography

## Abstract

Multiple sclerosis (MS) is a chronic neurological disease marked by demyelination, neurodegeneration, and widespread network dysfunction. While conventional MRI remains central to diagnosis, advanced techniques such as functional MRI (fMRI), electroencephalography (EEG), and electromyography (EMG) are increasingly recognized for their ability to capture dynamic functional changes that underlie clinical symptoms. This review explores the individual and combined applications of fMRI, EEG, and EMG in MS, emphasizing recent clinical findings from 2019 to 2024. fMRI provides high-resolution mapping of brain activation and connectivity, revealing compensatory plasticity in early stages and connectivity breakdowns associated with progression. EEG offers real-time monitoring of cortical activity, detecting spectral slowing, network reorganization, and neurophysiological correlates of fatigue and cognitive decline. EMG quantifies neuromuscular output, identifying spasticity, motor unit loss, and gait disturbances with high sensitivity. Integration of these modalities enhances spatial and temporal resolution; however, challenges such as data standardization and interpretive variability must be addressed to ensure robust biomarker development. Advances in machine learning, portable EEG/EMG systems, and big-data infrastructure are driving the translation of multimodal monitoring into clinical practice. Real-time assessments and individualized biomarker profiles could enable earlier diagnosis, more accurate prognosis, and personalized rehabilitation and therapy strategies. Although technical, interpretive, and standardization challenges remain, the convergence of fMRI, EEG, and EMG offers a promising path toward precision medicine in MS. Multimodal approaches not only deepen understanding of MS pathophysiology but also hold tangible potential to transform disease monitoring, treatment decision-making, and patient outcomes.

## Introduction

Multiple sclerosis (MS) is a chronic immune-mediated disorder of the central nervous system (CNS), marked by demyelination, axonal degeneration, and progressive neurological dysfunction [1]. Although conventional magnetic resonance imaging (MRI) has significantly improved diagnostic capabilities, it frequently fails to correlate with clinical symptoms and disability levels, highlighting a phenomenon known as the "clinico-radiological paradox" [2]. This discrepancy can partly be explained by the brain’s compensatory mechanisms, including functional reorganization that maintains performance despite structural damage, particularly in early stages of the disease [3]. As a result, there is increasing interest in integrating neuroimaging with neurophysiological assessments to provide a more comprehensive view of MS-related neural dysfunction [3][4]. Multimodal approaches that combine functional MRI (fMRI), electroencephalography (EEG), and electromyography (EMG) are particularly promising, as they offer complementary insights that may resolve the clinico-radiological paradox by elucidating mechanisms of functional compensation and disconnection [3].

fMRI has emerged as a valuable tool for assessing altered neural activity and connectivity in MS, particularly in regions that appear structurally unaffected [5]. Resting-state fMRI studies have revealed disruptions in large-scale functional networks, such as the default mode and sensorimotor networks, which correlate with both cognitive impairment and physical disability in MS patients [6][7]. These changes reflect a shift from adaptive neuroplasticity to maladaptive connectivity as the disease progresses [5]. fMRI is also increasingly used in longitudinal studies to monitor disease progression and evaluate the effectiveness of cognitive and motor rehabilitation programs [5].

On the other hand, EEG offers complementary information by directly measuring neuronal electrical activity with high temporal resolution. MS patients commonly exhibit EEG slowing, characterized by increased delta and theta activity and reduced alpha power, which is thought to result from widespread cortical disconnection [8]. Recent advances in quantitative EEG and network analysis have made it possible to identify biomarkers of cognitive decline and assess brain functional integrity in MS with greater precision [9]. Moreover, EEG-based metrics have demonstrated potential in predicting treatment outcomes and identifying patients at risk for rapid progression [10].

Moreover, EMG and related electrophysiological techniques, such as motor and sensory evoked potentials, play an essential role in evaluating the functional integrity of specific neural pathways [11]. In MS, delayed central conduction times and reduced amplitudes of motor evoked potentials are frequently observed and correlate strongly with motor disability [12]. Similarly, visual evoked potentials (VEPs) remain a sensitive tool for detecting optic pathway damage, often identifying abnormalities even in asymptomatic patients [13]. Multimodal evoked potential testing has proven especially useful in capturing subclinical impairments across multiple systems and holds promise for prognostic modeling in MS [14].

The integration of fMRI, EEG, and EMG findings offers a richer understanding of MS pathology and holds the potential to enhance clinical decision-making [3][15]. By combining these modalities, researchers and clinicians can gain a more nuanced view of CNS dysfunction, track disease evolution more accurately, and optimize therapeutic interventions [3]. This review aims to evaluate recent advances in multimodal imaging and electrophysiological approaches, focusing on their clinical relevance for diagnosis, monitoring, and treatment evaluation in MS.

## Pathophysiological Basis of Multiple Sclerosis (MS)

MS is an immune-mediated demyelinating disease of the brain and spinal cord characterized by focal plaques of myelin loss, axonal damage, and gliosis [16]. These neuropathological changes disrupt nerve conduction and lead to the varied neurological deficits observed in MS. Over time, the accumulation of irreversible tissue injury (especially axonal and neuronal loss) drives progressive disability [17]. Understanding the underlying pathology and its clinical correlates is crucial for developing biomarkers and therapies in MS.

### Neuropathological Features of MS Lesions

The neuropathological hallmark of MS is the presence of demyelinated plaques, which can be categorized into active, chronic active (smoldering), and chronic inactive lesions [18]. Active lesions are associated with ongoing inflammation, characterized by lymphocytic infiltration and myelin phagocytosis, while chronic active lesions exhibit a slowly expanding edge of activated microglia, suggesting persistent subclinical damage [18][19]. Chronic inactive lesions are fully demyelinated, gliotic, and lack significant immune cell infiltration [20].

Axonal injury, evident even in early disease stages, is now recognized as a major driver of progressive disability in MS [21]. This is exacerbated by failed or incomplete remyelination, particularly in chronic stages, where oligodendrocyte precursor cells are unable to fully restore myelin sheaths [22]. Remyelinated plaques, termed "shadow plaques", demonstrate thinner myelin but retain some conduction capacity and may confer neuroprotection [23].

MS lesions have a predilection for specific CNS regions, including the periventricular white matter, corpus callosum, juxtacortical areas, spinal cord, optic nerves, and infratentorial structures [24]. Periventricular lesions are classically aligned perpendicular to the lateral ventricles and are among the most specific features on MRI [2][19]. Cortical lesions, especially subpial demyelination, are increasingly recognized as clinically significant, particularly for cognitive impairment and fatigue [25][26].

Spinal cord lesions are strongly associated with motor dysfunction and sphincter disturbances. Cervical spinal cord atrophy, in particular, correlates with gait and mobility impairment, serving as a marker of disease progression [27]. Similarly, lesions in the optic nerves often clinically manifesting as optic neuritis can lead to visual impairment. Even after clinical recovery, delayed visual VEPs suggest persistent demyelination [13].

While inflammation dominates early MS, progressive forms are largely driven by neurodegeneration, including axonal transection, mitochondrial dysfunction, and neuronal loss [17]. These degenerative changes are accompanied by global and regional atrophy, particularly in the thalamus, cortex, and spinal cord [21][27]. Brain atrophy has emerged as a robust imaging biomarker, correlating with clinical worsening and cognitive decline [2].

One proposed mechanism for progressive neurodegeneration is compartmentalized inflammation, as seen in chronic active lesions where microglial activation persists despite the absence of blood-brain barrier breakdown [18]. Additionally, subpial cortical demyelination is believed to be driven by meningeal inflammation, which is particularly prominent in secondary progressive MS [25][26].

## fMRI in MS

### Resting-state vs Task-based fMRI in MS

fMRI can be performed either at rest or during specific tasks, each providing distinct insights. Task-based fMRI (tb-fMRI) measures brain activation while the patient performs a cognitive, motor, or sensory task, thus highlighting which regions and circuits are recruited for that function [6][28]. For example, MS patients often show altered activation of motor and cognitive regions during tasks compared to healthy controls, reflecting neuroplastic reorganization [28]. Early in the disease, tb-fMRI studies have noted increased activation in task-related regions (e.g. greater recruitment of motor or frontal areas) - interpreted as a beneficial compensatory response to damage - whereas in later stages, reduced activation is observed, correlating with higher lesion burden and clinical decline as compensatory mechanisms become exhausted [7][28]. In contrast, resting-state fMRI (rs-fMRI) maps spontaneous brain activity when the patient is not engaged in an active task, revealing the brain’s intrinsic functional connectivity networks [28]. An advantage of rs-fMRI is that it requires no patient participation; this avoids confounds from inability to perform tasks and allows assessment even in patients with severe disability [6]. Rs-fMRI also efficiently identifies multiple networks simultaneously (e.g. motor, visual, default-mode) in one scan, whereas task-based studies would require separate tasks for each [29]. Clinically, tb-fMRI is valuable for probing specific functional pathways (for instance, correlating motor cortex activation with hand function), while rs-fMRI excels at evaluating global network integrity and connectivity disruptions that underlie symptoms even when overt task performance is not being measured [30]. In practice, both approaches are complementary: task-based fMRI pinpoints how the brain tries to maintain function during challenges, and resting-state fMRI shows which networks are abnormally wired or synchronized at baseline. Together, they have advanced understanding of MS-related neuroplasticity and functional reorganization. Incorporating dynamic functional connectivity analyses could further refine these network-level biomarkers [6].

### Functional Connectivity Changes in MS

fMRI research has shown that MS disrupts functional connectivity, particularly in motor and cognitive networks [28][31]. Studies have revealed reduced sensorimotor connectivity and increased default mode network (DMN) centrality, particularly in patients with greater disability [31]. Graph-based analysis has identified disrupted global connectivity patterns in MS, correlating with cognitive performance [5]. Longitudinal studies indicate that network reorganization shifts from adaptive hyperconnectivity in early disease to a collapse of network efficiency in advanced MS [28][32]. In highly disabled patients, increased connectivity within the sensorimotor cortex has been reported, possibly reflecting maladaptive plasticity or an exhausted compensatory response [33].

### fMRI in MS Fatigue

Fatigue is one of the most prevalent symptoms in MS, and fMRI studies have provided insight into its neural correlates [34]. Resting-state fMRI studies have found increased connectivity in the DMN among fatigued patients, suggesting altered resting-state modulation [35]. Dynamic connectivity analyses have shown reduced variability in the basal ganglia and attentional networks, potentially underlying mental exhaustion and effort intolerance [36]. Task-based fMRI supports these findings, demonstrating reduced recruitment of typical task-relevant areas and increased compensatory activity in others, such as the frontal cortex [29].

### fMRI and Cognitive Impairment in MS

Cognitive impairment in MS is associated with disrupted functional connectivity, particularly in the DMN and frontoparietal networks [28][32]. Graph theoretical studies indicate that decreased hub integrity and efficiency within these networks relate to slower cognitive processing [37]. These alterations align with clinical assessments, such as the Symbol Digit Modalities Test (SDMT), suggesting that fMRI markers may serve as early indicators of cognitive deterioration [31].

### fMRI and Motor Dysfunction in MS

Task-based fMRI studies show altered recruitment of motor networks in MS, with increased bilateral activation and enhanced cerebellar involvement during movement tasks[7]. In early MS, this may represent compensatory mechanisms. However, as disability progresses, decreased motor network efficiency and reduced task-related activation are observed [33]. Resting-state fMRI studies further support these findings, showing that sensorimotor network efficiency changes correlate with motor impairment levels, independent of lesion load[6][29][30]. Altered cerebellar and basal ganglia connectivity has also been linked with symptoms such as tremor and gait instability [38].

## Electroencephalography in MS

### Evoked Potentials in MS

Evoked potentials (EPs) provide objective measures of signal conduction in central nervous system pathways and frequently uncover subclinical demyelination in MS [14].

Visual evoked potentials (VEP): MS patients often show prolonged P100 latency, reflecting demyelination of the optic nerves. VEP abnormalities can be detected even in asymptomatic eyes, making them valuable for diagnosis and monitoring of optic pathway involvement [13].

Somatosensory evoked potentials (SEP): SEP abnormalities due to dorsal column or brainstem lesions are also common in MS and often precede clinical symptoms [14].

Motor evoked potentials (MEP): These assess corticospinal tract integrity using transcranial magnetic stimulation. MEP abnormalities correlate with pyramidal tract dysfunction and are more pronounced in progressive forms of MS [12].

P300 potentials: The P300 wave is often delayed and reduced in amplitude in MS patients, indicating impaired cognitive processing [39]. Longer P300 latencies correlate with reduced performance on neuropsychological testing and have been shown to predict long-term disability accumulation [40].

Composite EP score integrating VEP, SEP, and MEP findings are increasingly used as biomarkers to track global CNS damage and have shown predictive value for future disability [41].

### Resting-state EEG Oscillatory Changes

Resting EEG recordings in MS frequently show diffuse slowing of brain rhythms. The posterior dominant rhythm, typically in the alpha range (8-12 Hz), is often shifted toward lower frequencies in MS patients, particularly those with more advanced disease [8]. Increased theta and delta activity and reduced alpha/beta power have been associated with cortical demyelination and global brain atrophy [9]. Quantitative EEG analyses link spectral changes to cognitive impairment; however, high inter-subject variability and inconsistent study protocols limit generalizability and warrant standardized methodologies [10].

### Network Dysfunction and Neuroinflammation

EEG also captures changes in large-scale network dynamics. In progressive MS, studies have shown decreased inter-regional coherence particularly in alpha and theta bands which correlates with lesion burden and cognitive decline [9]. This desynchronization reflects the functional consequences of structural disconnection caused by widespread demyelination and neuroinflammation. Inflammatory activity is thought to disrupt thalamo-cortical and cortico-cortical interactions, which are visible in EEG as increased slow-wave activity and reduced higher-frequency synchrony [36].

Notably, EEG abnormalities are also seen in MS patients with epilepsy ,a condition more prevalent in MS than in the general population, suggesting that cortical demyelination can lead to hyperexcitability and network instability [8].

### Clinical Applications of EEG in MS

Given these neurophysiological signatures, EEG-based techniques have multiple clinical applications in MS management and research:

Adjunct to Diagnosis: Evoked potentials, especially VEP, are used to provide objective evidence of CNS lesions as part of MS diagnosis. A prolonged P100 latency on VEP can confirm a past optic neuritis even if MRI is normal [14][39]. Recent studies have proposed formally adding the optic nerve (assessed by VEP) as a fifth region to the McDonald diagnostic criteria for dissemination in space, which slightly improves diagnostic sensitivity [24][42]

Monitoring disease activity: measurements over time can help monitor MS progression. Changes in EP latencies may indicate new or worsening demyelination even between clinical relapses. Multimodal EP scores have shown promise for tracking disease severity [41]. Because EEG is low-risk and inexpensive, serial EP testing could be used as a practical adjunct to MRI for monitoring MS, especially in settings where frequent MRI is impractical[41][43]. Some have even applied machine learning to EP data and found that EP-based models can predict disability progression with accuracy approaching that of MRI-based models [43].

Cognitive Assessment and Prognostication:

Prolongation of the P300 latency, in particular, correlates with cognitive impairment at a single time-point and also has prognostic significance. Patients with markedly delayed P300 are more likely to develop severe cognitive disability over the long term [44].

Therapeutic Response Prediction: Improvements in EP latency may serve as an objective sign of CNS functional recovery (e.g. through remyelination) in response to therapies. For example, in trials of experimental remyelinating agents, shortening of VEP P100 latency is taken as evidence of repair in the optic nerve [45].

Moreover, A study evaluating autologous hematopoietic stem cell transplant (AHSCT) in MS noted that while VEP/SEP latencies on average remained unchanged, a subset of patients demonstrated improved conduction velocity in certain pathways post-transplant [46]. Beyond pharmacologic treatments, EEG is being explored in neurorehabilitation; for instance, neurofeedback therapy in MS aims to train patients to modify their brain rhythms (e.g. reduce abnormal beta coherence) to alleviate fatigue or cognitive symptoms [47].

## Electromyography in MS

### Surface and Needle EMG in Spasticity and Motor Unit Function

Surface EMG (sEMG) is a noninvasive technique used to measure muscle activity noninvasively for the evaluation of neurologic disorders that is plays a key role in quantifying spasticity in MS [48][49]. It detects abnormal muscle activation patterns during passive stretch, including exaggerated dynamic and static stretch reflexes also it exaggerated velocity-dependent responses (DSR) and resting muscle activity (spastic dystonia) in hypertonic MS muscle [48]. On the other hand, needle EMG is typically normal in MS because peripheral nerves and muscles are not primarily affected [50]. However, it may show reduced motor unit recruitment or subtle disuse changes in severely impaired muscles [51]. Surface EMG, when combined with gait kinematics from motion sensors or video analysis, provides deeper insights into compensatory movement patterns during rehabilitation [52].

### EMG Insights into MS Fatigue and Gait Disturbances

MS patients exhibit declining EMG amplitude (RMS) during sustained contractions, whereas healthy controls typically show increased amplitude due to progressive motor unit recruitment [50]. This paradoxical decrease indicates central fatigability and poor neuromuscular drive in MS [50]. Similarly, Eken et al. [53] showed that after treadmill walking, MS patients had greater EMG median frequency decline and increased amplitude in the calf muscles, confirming greater local muscle fatigue during gait.

### EMG in Gait Abnormalities

Patients who are suffering MS, exhibit widened overlapping muscle synergies during gait, likely representing a compensatory strategy to maintain locomotion despite CNS damage [54]. This increased "fuzziness" of motor activation was more pronounced in patients with worse balance [54][55]. sEMG also identifies specific deficits such as delayed plantar flexor activation and reduced ankle push-off, which correlate with reduced gait speed and stride length [51]. These findings have been applied clinically to guide personalized rehabilitation strategies [51][52].

## Multimodal Integration of fMRI, EEG, and EMG in MS

**Table:** Table1. Comparison of fMRI, EEG, and EMG Modalities in MS

**Feature**	fMRI	EEG	EMG
Primary Domain	Brain hemodynamics and network connectivity	Cortical electrical activity	Muscle activation and neuromuscular output
Clinical Targets in MS	Cognitive dysfunction, fatigue, motor reorganization	Cognitive decline, fatigue, network dysregulation	Spasticity, fatigue, gait, peripheral motor assessment
Portability	Low	Moderate to high	High (with wearable EMG systems)
Use in Multimodal Fusion	Provides spatial anchor	Adds timing and dynamic modulation	Links brain activity to muscle output

### Complementary Strengths and Resolution Trade-offs

Each modality offers unique advantages in studying MS, and combining them leverages their complementary strengths. fMRI provides high spatial resolution (millimeter-scale mapping of brain activity) but low temporal resolution, since the blood-oxygen-level-dependent signal unfolds over seconds [28]. In contrast, EEG directly measures neuronal electrical activity with millisecond temporal resolution, though its spatial localization is limited by signal mixing across the scalp [9][56]. Table-1 shows a comparison of fMRI, EEG, and EMG modalities.

### Synchronization and Data Fusion Strategies

Simultaneous EEG-fMRI requires synchronization of hardware clocks and artifact correction algorithms to manage MRI-induced noise in EEG signals [57]. Strategies such as EEG-informed fMRI allow time-locked EEG features to guide BOLD signal analysis, while fMRI-informed EEG enhances source localization using anatomical priors [33][58]. More advanced approaches, like joint ICA or machine learning, extract shared components or patterns across modalities, offering deeper insights into MS network dysfunction [3]. Cortico-muscular coherence analysis links EEG and EMG to quantify motor coupling; distinguishing simultaneous from sequential modality integration would clarify technical and interpretive challenges [59].

## Recent Multimodal Studies in MS

EEG-fMRI integration: Baldini et al. [60] found altered EEG microstates in MS patients, reflecting changes in resting-state network dynamics typically studied by fMRI. Also, Shin et al. [61] conducted an fMRI study of visual cortex activity in MS with concurrent EEG and a hypercapnia (CO2) challenge to probe neurovascular reactivity. By measuring EEG alongside fMRI during visual stimulation, they aimed to discern whether differences in fMRI activation between MS patients and healthy controls were due to impaired neurovascular coupling or neural activity changes [61].

EEG-EMG integration for motor dysfunction: MS frequently impairs motor pathways, and researchers have combined EEG and EMG to study this. Tomasevic et al. [59] demonstrated that MS fatigue correlates with shifts in EEG-EMG coherence frequency, predicting over 65% of fatigue severity variance. This suggests brain-muscle desynchronization as an early biomarker [59].

Moreover, Resting-state EEG connectivity measures, such as alpha-band phase lag index (wPLI) and symbolic mutual information (wSMI), can predict and track improvements in motor performance following intensive rehabilitation in MS. These measures may help customize and optimize rehabilitative interventions [62].

Trimodal integration (fMRI, EEG, EMG) in motor fatigue: A cutting-edge approach by Leodori et al. [63] illustrates the power of combining all three modalities. In their multimodal study, they investigated the neural bases of motor fatigue in MS using transcranial magnetic stimulation (TMS) coupled with EEG and EMG. They assessed patients before and after administration of natalizumab to see how fatigue "wearing-off" correlated with neurophysiological changes [63][64]. Such multimodal evidence also helps validate fatigue as a real physiological phenomenon in MS, not just subjective, and provides targets for intervention [64].

Multimodal MRI plus EEG outcomes prediction: Other clinical studies have used EEG alongside MRI measures to predict patient outcomes [65][66]. An illustrative example is an observational study where MS patients underwent rehabilitative motor training and researchers measured both MRI lesion load and EEG connectivity before the therapy [62]. Interestingly, EEG-based functional connectivity (specifically, resting-state EEG coherence measures in the alpha band) was found to predict which patients would show the most improvement after training, whereas conventional MRI lesion load did not predict improvement [62][66].

## Challenges and Limitations

**Table:** Table2. The Strengths and Limitations of fMRI, EEG, and EMG Modalities

**Modality**	**Strengths**	**Limitations**
**fMRI**	High spatial resolution; detects functional reorganization and brain connectivity changes; useful in cognitive and motor network mapping	Expensive; low temporal resolution; sensitive to motion and physiological noise; complex data interpretation
**EEG**	High temporal resolution; captures cortical oscillations and evoked potentials; portable and relatively affordable	Low spatial resolution; signal contamination (e.g., muscle, eye artifacts); requires trained interpretation
**EMG**	Direct measure of neuromuscular output; sensitive to spasticity, muscle fatigue, and motor unit recruitment; high temporal resolution	Limited insight into central nervous system; surface EMG affected by skin-electrode contact and crosstalk; needle EMG is invasive

Despite the growing interest in using advanced neurophysiological tools to support MS management, several challenges limit their integration into clinical practice [67]. These include technical constraints, interpretative complexity, high costs, and a lack of standardized protocols across centers [15][66][67].Table-2 shows the strengths and limitations of modalities.

Technical limitations begin at the point of data acquisition. fMRI requires high-field MRI scanners, strict motion control, and dedicated sequences factors that make the technique highly sensitive to physiological artifacts and patient movement [68]. Differences in scanner field strength, acquisition parameters, and preprocessing pipelines further reduce reproducibility and comparability across clinical sites [7][28]. EEG and sEMG, while less expensive and more portable, are prone to signal contamination from muscle artifacts, environmental noise, and inconsistent electrode placement [47][50]. In both modalities, the quality of the recording is highly dependent on the operator's experience, environment control, and equipment maintenance [69].

Interpretation issues hinder clinical adoption; distinguishing research-based complexities from clinical interpretation barriers (e.g., standard reporting tools) would sharpen this critique. fMRI data requires advanced post-processing and trained neuroimaging experts to interpret subtle activation or connectivity patterns [70]. Clinical heterogeneity in MS adds a layer of complexity what may appear as hyperactivation in one patient could reflect compensation, while in another, it may be a marker of maladaptive plasticity [7]. In EEG and EMG, even skilled neurologists often require additional neurophysiology training to confidently interpret abnormal rhythmicity, evoked potentials, or fatigability patterns [71]. A survey by Manca et al. [72] showed that 97% of neurorehabilitation specialists reported difficulty using surface EMG clinically without specialized education. Similarly, variations in EMG and EEG signal analysis approaches (e.g., filter settings, epoch lengths, frequency bands) significantly impact data interpretation and diagnostic conclusions [69].

Cost and accessibility are persistent barriers. fMRI remains prohibitively expensive for routine use, particularly in outpatient neurology clinics or in low-resource settings. The infrastructure costs associated with MRI hardware, maintenance, and specialized personnel restrict its use largely to academic centers [73]. While EEG and EMG are more affordable and portable, they still require trained technicians, setup time, and equipment upkeep. In practice, many facilities avoid deploying these tools due to lack of reimbursement or perceived logistical burden [72].

## Future Directions

The coming years promise a convergence of multimodal neurophysiological data with artificial intelligence (AI), portable technologies, and large-scale digital infrastructure in MS [74][75]. Supervised classifiers such as random forests have integrated EEG coherence and fMRI connectivity to predict Expanded Disability Status Scale (EDSS) progression with 85% accuracy[43]. Similarly, multimodal feature sets have been shown to improve disability prediction in MS when compared to single-modality inputs alone [76].

On the other hand, Portable and wearable technologies are also shifting the landscape. Low-field mobile MRI systems have recently demonstrated the ability to detect MS lesions with high sensitivity, offering a potential avenue for bedside or community-based imaging [77]. In parallel, lightweight and wireless EEG and EMG systems are enabling the capture of brain and muscle signals in naturalistic settings [78]. Surface EMG and inertial sensors have already been used to monitor spasticity and gait disturbances outside the clinic, with real-world measures correlating well with clinical disability [79]. Such tools may one day facilitate home-based neurophysiological monitoring, potentially enabling earlier intervention when symptoms subtly worsen [78].

## Conclusion

This integrative review highlights the pivotal role of multimodal approaches, combining functional magnetic resonance imaging (fMRI), electroencephalography (EEG), and electromyography (EMG), in advancing the understanding and management of multiple sclerosis (MS). fMRI provides detailed mapping of functional connectivity and compensatory neural networks. EEG captures oscillatory changes and evoked potentials indicative of cognitive decline and fatigue. EMG offers a quantitative assessment of spasticity, muscle fatigue, and gait disturbances. Together, these modalities provide complementary insights into the central and peripheral pathophysiology of MS, addressing the clinico-radiological paradox. Recent studies, such as Leodori et al. [63], demonstrate the power of integrating TMS-EEG-EMG to elucidate motor fatigue mechanisms, while Baldini et al. [60] highlight EEG microstates as markers of altered large-scale network dynamics. These multimodal approaches enhance early diagnosis, predict disease progression, and monitor responses to innovative therapies, such as remyelinating agents or neurorehabilitation, with robust correlations to clinical metrics like the Expanded Disability Status Scale (EDSS).

Despite these advances, challenges, including data standardization, susceptibility to artifacts, and high costs, limit widespread clinical adoption. Emerging technologies, such as portable EEG/EMG systems, low-field MRI, and machine learning algorithms (e.g., predicting EDSS progression with high accuracy), hold promise for overcoming these barriers, enabling real-time monitoring and personalized medicine in MS.

Ultimately, this review emphasizes that the convergence of neuroimaging and electrophysiological modalities not only deepens the mechanistic understanding of compensatory and degenerative processes in MS but also offers transformative potential for diagnosis, prognosis, and therapeutic decision-making. Future research should prioritize large-scale longitudinal studies, integration of artificial intelligence for complex data analysis, and evaluation of these biomarkers’ impact on patient outcomes to drive MS management toward a precise, data-driven, and patient-centered paradigm. These advancements pave the way for interdisciplinary research and may significantly alleviate the global burden of MS.

## Conflict of Interest

The author declares that the research was conducted in the absence of any commercial or financial relationships that could be construed as a potential conflict of interest.
